# Assessment of BoHV-4-based vector vaccine intranasally administered in a hamster challenge model of lung disease

**DOI:** 10.3389/fimmu.2023.1197649

**Published:** 2023-07-06

**Authors:** Seok-Chan Park, Laura Conti, Valentina Franceschi, Byungkwan Oh, Myeon-Sik Yang, Gaeul Ham, Antonino Di Lorenzo, Elisabetta Bolli, Federica Cavallo, Bumseok Kim, Gaetano Donofrio

**Affiliations:** ^1^ Biosafety Research Institute and Laboratory of Veterinary Pathology, College of Veterinary Medicine, Jeonbuk National University, Iksan, Republic of Korea; ^2^ Department of Molecular Biotechnology and Health Sciences, University of Torino, Torino, Italy; ^3^ Department of Medical Veterinary Sciences, University of Parma, Parma, Italy

**Keywords:** vaccine, lung disease, BoHV-4-based vector, intranasal vaccination, immune response

## Abstract

**Introduction:**

Bovine herpesvirus 4 (BoHV-4) is a bovine *Rhadinovirus* not associated with a specific pathological lesion or disease and experimentally employed as a viral vector vaccine. BoHV-4-based vector (BoHV-4-BV) has been shown to be effective in immunizing and protecting several animal species when systemically administrated through intramuscular, subcutaneous, intravenous, or intraperitoneal routes. However, whether BoHV-4-BV affords respiratory disease protection when administered intranasally has never been tested.

**Methods:**

In the present study, recombinant BoHV-4, BoHV-4-A-S-ΔRS-HA-ΔTK, was constructed to deliver an expression cassette for the SARS-CoV-2 spike glycoprotein, and its immunogenicity, as well as its capability to transduce cells of the respiratory tract, were tested in mice. The well-established COVID-19/Syrian hamster model was adopted to test the efficacy of intranasally administered BoHV-4-A-S-ΔRS-HA-ΔTK in protecting against a SARS-CoV-2 challenge.

**Results:**

The intranasal administration of BoHV-4-A-S-ΔRS-HA-ΔTK elicited protection against SARS-CoV-2, with improved clinical signs, including significant reductions in body weight loss, significant reductions in viral load in the trachea and lungs, and significant reductions in histopathologic lung lesions compared to BoHV-4-A-S-ΔRS-HA-ΔTK administered intramuscularly.

**Discussion:**

These results suggested that intranasal immunization with BoHV-4-BV induced protective immunity and that BoHV-4-BV could be a potential vaccine platform for the protection of other animal species against respiratory diseases.

## 1 Introduction

BoHV-4 belongs to the *Rhadinovirus* genus, *γ-Herpesviridae* subfamily, and Herpesviridae family. BoHV-4 has been isolated mainly from healthy cattle. Most γ-herpesviruses strictly replicate in their natural host species. However, BoHV-4 is able to replicate in different host species either *in vivo* or *in vitro* (1). In addition to cattle, BoHV-4 has been isolated from other ruminants, and occasional isolations were obtained in lions, cats, and healthy owl monkeys (*Aotus trivirgatus*) (1). Taken together, the molecular and biological characteristics of BoHV-4 make it a good candidate for a viral vaccine vector (1). The virus has little or no pathogenicity, no oncogenicity, the capability to accommodate large amounts of foreign genetic material, the ability to be maintained in an episomal state in dividing cells (2), a broad host range with the ability to infect several cell types from different animal species, and the ability to maintain transgene expression during differentiation (1). Importantly, the BoHV-4 strain utilized was derived from a cell fraction of milk obtained from a healthy cow. The entire BoHV-4 genome was sequenced and cloned as a bacterial artificial chromosome to allow molecular manipulation to create a vector platform (3–5). *In vitro*, BoHV-4 can replicate in primary cell cultures or cell lines from a broad spectrum of host species, including sheep, goats, swine, cats, dogs, rabbits, mink, horses, turkeys, ferrets, chickens, hamsters, rats, mice, monkeys, and humans (1). Experimentally, recombinant BoHV-4 delivering heterologous antigens immunized rodents (mice and rats) (6–8), rabbits (9, 10), chickens (11), sheep (12), goats (13), and pigs (14). We previously demonstrated that BoHV-4 was an effective vaccine platform for both infectious disease prevention and cancer therapy when administered systemically through intramuscular, subcutaneous, or intraperitoneal routes in experimental models and veterinary clinical trials (8, 13, 15–18). However, we believe that it could also represent a valuable tool for intranasal vaccination against respiratory diseases. Because the COVID-19 pandemic has generated a lot of interest in terms of vaccination strategies against agents inducing lung pathology and several laboratory animal models have been established (19), COVID-19 was chosen as the disease model to test the efficacy of intranasally delivered BoHV-4-based vector (BoHV-4-BV). SARS-CoV-2 Spike (S) glycoprotein is a key factor in SARS-CoV-2 cell infection, largely exploited for vaccine generation against COVID-19, and the serum anti-Spike neutralizing antibody titer is considered to correlate with protection. For these reasons, recombinant BoHV-4 was constructed to deliver SARS-CoV-2 Spike and tested in a preclinical model (19). Since mice are not the ideal model for studying SARS-CoV-2-induced disease as mouse ACE2 poorly binds to SARS-CoV-2 Spike protein, the Syrian hamster model was selected. The sensitivity of hamsters to SARS-CoV-2 infection was previously demonstrated. The histopathological lesions induced in the respiratory tracts of SARS-CoV-2-infected animals were previously extensively characterized and resembled the lesions observed in human patients (20, 21). The intranasal inoculation of hamsters with SARS-CoV-2 results in the infection of the upper and lower respiratory tracts, with maximal viral replication three days after infection and damage to the respiratory epithelium in the trachea and lungs, accompanied by alveolar edema, bronchopneumonia, interstitial pneumonia, and immune cell infiltration (21, 22). Clinically, the infection causes significant weight loss and respiratory distress, making this model a good tool for studying vaccine efficacy in respiratory diseases (19, 21, 22). Therefore, we compared the intranasal versus intramuscular administration of BoHV-4-BV expressing the SARS-CoV-2 Spike protein in both BALB/c mice, to characterize the immune response, and Syrian hamsters, demonstrating that vaccination induced the production of neutralizing antibodies independent of the delivery route. However, intranasal administration was superior in inducing viral clearance from the respiratory tract and reducing disease symptoms, demonstrating that BoHV-4 is a promising tool for the development of new intranasal vaccines to prevent respiratory infections.

## 2 Materials and methods

### 2.1 Cells

Bovine embryonic kidney (BEK) cells (Istituto Zooprofilattico Sperimentale, Brescia, Italy; BS CL-94), BEK *cre*, expressing cre recombinase (5), human embryonic kidney cells (HEK) 293T (ATCC: CRL-11268), and Madin-Darby bovine kidney (MDBK) cells (ATCC: CRL 6071) were grown in complete Eagle’s minimal essential medium (cEMEM: 1 mM sodium pyruvate, 2 mM of L-glutamine, 100 IU/mL of penicillin, 100 μg/mL of streptomycin, and 0.25 μg/mL of amphotericin B), supplemented with 10% fetal bovine serum (FBS), and incubated at 37°C and 5% CO_2_ in a humidified incubator. All supplements for the culture medium were purchased from Gibco. Stably transfected HEK/ACE2/TMPRRS2/Puro cells were obtained as previously described (23) and maintained in complete EMEM with 10% FBS supplemented with 2 µg/mL of puromycin (Millipore Merck Life Science).

### 2.2 Transient transfection

HEK293T cells were seeded into 25 cm^2^ flasks (1 × 10^6^ cells/flask) and were transfected with pINT2-(TK-EF1α-S-ΔRS-HA-TK) or pEGFP-C1 (mock control) using polyethyleneimine (PEI) transfection reagent (Polysciences, Inc.). S-ΔRS-HA ORF was chemically synthesized by Eurofins genomics (Milano, Italy) and integrated into a pINT2 transfer vector (1) to yield pINT2-(TK-EF1α-S-ΔRS-HA-TK). pEGFP-C1 was obtained from Clontech. HEK293T cells transfection was performed as previously described (23). For syncytia formation, HEK/ACE2/TMPRRS2/Puro cells were transiently cotransfected with pINT2-(TK-EF1α-S-ΔRS-HA-TK) and pEGFP-C1 plasmids at the same molar ratio (1:1), using PEI transfection reagent as described before. Twenty-four hours after co-transfection, syncytia were observed by inverted fluorescence microscopy (Zeiss-Axiovert-S100), and images were acquired by a digital camera (Zeiss-Axiocam-MRC).

### 2.3 BAC recombineering and selection and southern blotting

Recombineering was performed as previously described with some modifications. The expression vector pINT2-(TK-EF1α-S-ΔRS-HA-TK) was PvuI-linearized and electroporated in heat-induced SW102 *Escherichia coli* (*E. coli*) containing pBAC-BoHV-4-A-KanaGalK-ΔTK (5) to obtain pBAC-BoHV-4-A-S-ΔRS-HA-ΔTK.

pBAC-BoHV-4-A-S-ΔRS-HA-ΔTK DNA was purified and analyzed through HindIII restriction enzyme analysis and Southern Blotting with a specific probe labeled with digoxigenin. The probe was generated by polymerase chain reaction (PCR) with S450 sense (ATG TTC GTG TTC CTG GTG CTG CTG) and S450 antisense (CTT GTT GTT CTT GTG GTA GTA CAC GCC) primers. A detailed protocol can be found in a previously published paper (5).

### 2.4 Cell culture electroporation and recombinant virus reconstitution

BEK or BEK *cre* cells were maintained as a monolayer in cEMEM growth medium with 10% FBS. pBAC-BoHV-4-A and pBAC-BoHV-4-A-S-ΔRS-HA-ΔTK DNA (approximately 5 µg) were electroporated (Biorad, Gene Pulser XCell, 270 V, 1500 µF, 4-mm gap cuvettes) in 600 µL of high-glucose DMEM without serum (Euroclone) into BEK and BEK *cre* cells from a confluent 25 cm^2^ flask. The electroporated cells were transferred to new flasks and incubated at 37°C with 5% CO_2_ overnight. Twenty-four hours after transfection, the medium was replaced with fresh cEMEM, and the cells were split 1:2 when they reached confluence 2 days post-electroporation. The cells were grown until the appearance of cytopathic effects (CPEs).

### 2.5 Viruses and viral replication

BoHV-4-A, BoHV-4-A-S-ΔRS-HA-ΔTK, BoHV-4-A-CMV-A29, and BoHV-4-A-CMVlucΔTK were propagated and titered as previously described (16).

### 2.6 Immunoblotting

Western immunoblotting analysis was performed on protein cell extracts from 25 cm^2^ flasks of HEK293 T cells transfected with pINT2-(TK-EF1α-S-ΔRS-HA-TK) or mock-transfected. Western immunoblotting was also performed with protein cell extracts obtained from BEK cells infected with 1 MOI of BoHV-4-A or BoHV-4-A-S-ΔRS-HA-ΔTK (23). Protein detection was made with a primary mouse monoclonal anti-HA tag antibody (G036, Abcam Inc.) diluted 1:10,000, probed with horseradish peroxidase (HRP)-labeled anti-mouse immunoglobulin (A9044, Sigma), diluted 1:15,000, and visualized by enhanced chemiluminescence (Clarity Max Western ECL Substrate, Bio-Rad).

### 2.7 SARS-CoV-2 pseudovirus generation and seroneutralization assay

Lentiviral vector-based SARS-CoV-2 Spike pseudoviruses were generated as previously described (23). Five different SARS-CoV-2 Spike pseudoviruses were produced, displaying Spike glycoproteins on their surface belonging to Wuhan-Hu-1 (B.1 Lineage; China), Alpha (B.1.1.7. Lineage; United Kingdom), Beta (B.1.351 Lineage; South Africa), Gamma (P.1 Lineage; Brazil), or Omicron (B.1.1.529 Lineage; Europe) variants (24). Heat-inactivated murine sera samples were tested at dilutions of 1:4, 1:8, 1:16, 1:32, 1:64, 1:128, 1:256, and 1:512. A negative control was established without serum. The complete method was fully described and published previously (23). The relative luciferase units (RLUs) were compared and normalized to those derived from wells where pseudovirus was added in the absence of sera (100%). Neutralization titer 50 (NT50) was expressed as the maximal dilution of sera with a signal reduction of ≥ 50%. Each serum was tested in triplicate.

### 2.8 Assessment of antibody levels in mice after vaccination

Murine sera samples were tested for SARS-CoV-2-specific IgG antibodies using a commercial quantitative two-step enzyme-linked immunosorbent assay (ELISA) (COVID-SeroIndex, Kantaro Quantitative SARS-CoV-2 IgG Antibody Kit, R&D Systems), according to the manufacturer’s recommendations. In this specific case of testing murine sera, an anti-mouse IgG-HRP-conjugated (A9044, Sigma) antibody, diluted 1:15,000 was used. All the animal sera were collected at T0, T1, and T2, prior to animal euthanization and were tested at a dilution of 1:10. (See paragraph 2.9).

### 2.9 Assessment of BoHV-4-A-S-ΔRS-HA-ΔTK immunogenicity in mice

Female BALB/c and severe combined immunodeficiency disease (SCID) mice were housed at the Molecular Biotechnology Center at the University of Torino following the European guidelines, Directive 2010/63, and with the approval of the Animal Care and Use Committee of the University of Torino and the Italian Ministry of Health. The mice (n = 5 per group) were vaccinated twice with injections of 200 µL intraperitoneally of DMEM containing 10^6^ TCID_50_ of BoHV-4-A-S-ΔRS-HA-ΔTK or BoHV-4-A-A29 (T0) and boosted 2 weeks apart (T1). Blood was collected from both groups of mice by retro-orbital bleeding at T0 and T1. Mice were euthanized 2 weeks from T1 (T2), and blood and spleens were collected. Blood was let to coagulate for 30 minutes at room temperature. Coagulated blood was then centrifuged at 3000 rpm for 10 minutes, and serum was collected and stored at -20°C. Spleens were smashed and passed through a 70-µm pore cell strainer, centrifuged at 1400 rpm for 10 minutes, and incubated in erythrocyte lysing buffer (155 mM NH_4_Cl, 15.8 mM Na_2_CO_3_, 1 mM EDTA, pH 7.3) for 10 minutes at room temperature. After washing in RPMI-1640 with 10% FBS, 20 x 10^6^ cells were resuspended in FBS with 10% DMSO (Sigma-Aldrich) and stored at -80°C until use.

### 2.10 Cytotoxicity

NIH/3T3 mouse fibroblasts stably transfected with H-2K^d^ and B7.1 (3T3/KB) (8b) were cultured in DMEM (Thermo Fisher Scientific) with 20% FBS. Cells were transfected with the pINT2-(TK-EF1α-S-ΔRS-HA-TK) plasmid using Lipofectamine 2000 according to the manufacturer’s instructions (25). Spike-expressing or non-transfected 3T3/KB target cells (10^4^) were incubated with 2 µM CFSE (Molecular Probes, cod. C34554) for 20 minutes at 37°C and then cultured with splenocytes at different effector:target (E:T) ratios for 48 hours as previously described (18). The cytotoxicity of non-transfected 3T3/KB target cells was analyzed as controls.

### 2.11 Hamster vaccination and infection

Five-week-old male Syrian hamsters (*Mesocricetus auratus*) were purchased from Central Lab Animals. Animal procedures, including challenging and necropsy, were performed in an animal biosafety level 3 (ABL3) facility of the Korea Zoonosis Research Institute (KOZRI) at Jeonbuk National University, with approval from the Institutional Animal Care and Use Committee (JBNU 2022-049). Hamsters were divided into four groups (n = 6 per group) based on vaccination (vaccination/control) and inoculation route (intramuscular (IM)/intranasal (IN)): (1) IM/BoHV-4-A-S-ΔRS-HA-ΔTK (IM-BoHV4-Spike) group, (2) IM-BoHV-4-A (IM-BoHV4-control), (3) IN/BoHV-4-A-S-ΔRS-HA-ΔTK (IN-BoHV4-Spike), and (4)IN-BoHV-4-A (IN-BoHV4-control) group. The IM-BoHV4-Spike and IN-BoHV4-Spike groups were vaccinated twice by inoculations of 200 µL (200 µL IM or 200 µL IN) of DMEM containing 10^6^ TCID_50_ of BoHV-4-A-S-ΔRS-HA-ΔTK and boosted 2 weeks apart (**Figure 4A**). IM-BoHV4-control and IN-BoHV4-control were vaccinated with BoHV-4-A by IM and IN inoculation, respectively, on the same schedule as the vaccinated group. IM vaccines were inoculated into the *quadriceps femoris* muscle of the left and right legs of each hamster (100 μL per leg for a total of 200 μL per hamster). IN vaccinations were inoculated by pipette (100 μL per nostril for a total of 200 μL per hamster), and the hamsters were anesthetized with isoflurane. Six weeks after the first vaccination, all hamsters were challenged by IN inoculations of SARS-CoV-2 (NCCP43326, Wuhan strain) at about 4 x 10^5^ TCID_50_ per hamster in 200 μL (100 μL per nostril). Following infection, all hamsters were clinically monitored daily and euthanized 3 days post-infection (dpi) (n = 3 per group) or days post-infection in dpi (n = 3 per group). Tracheas and lungs were collected for viral load quantification or histopathologic analysis.

### 2.12 RNA extraction and real-time PCR

Total RNA was isolated using Hybrid-R™ (GeneAll). The RNA was reverse transcribed using ReverTra Ace qPCR RT Master Mix (TOYOBO) to generate cDNA. SARS-CoV-2 receptor binding domain (RBD) gRNA expression and N subgenomic mRNA (sgmRNA) was determined using the following primers. The primers used for RBD gRNA were 5’-GCTCCATGGCCTAATATTACAAACTTGTGCC-3’ (forward), 5’-TGCTCTAGACTCAAGTGTCTGTGGATCAC-3’ (reverse), and for N sgmRNA 5’-CGATCTCTTGTAGATCTGTTC TCT-3’ (forward), 5’-TCTGGTTACTGCCAGTTGAATCTG-3’ (reverse). SARS-CoV-2 viral burden in tissues was quantified by qPCR relative to hamster HPRT expression (forward, 5’- TGCGGATGATATCTCAACTTTAACTG; reverse, 5’- AAAGGAAAGCAAAGTTTGTATTGTCA-3’). qPCR was performed using CFX96 Real-time PCR (Bio-Rad Laboratories) with qPCR SYBR Green Mix (PCRbiosystems).

### 2.13 Histopathology and scoring criteria

The collected tissues were fixed with 10% neutral phosphate-buffered formalin and processed and embedded in paraffin. The formalin-fixed paraffin-embedded tissue blocks were sectioned at 6 μm thicknesses using a microtome (HM-340E, Thermo Fisher Scientific Inc.,). Tissue sections were stained with hematoxylin and eosin (H&E) according to the standard protocol. Staining was scored by a slight modification of previously described scoring criteria (21). Briefly, the tracheas were scored according to three criteria: 1, damage of tracheal epithelial cells; 2. inflammatory cell infiltration of the lamina propria; and 3, exudates in the tracheal lumen. The lungs were scored according to four criteria: 1) inflammatory cell infiltration of the peribronchiolar or perivascular region; 2) exudates in the bronchiolar lumen; 3) damage to the bronchiolar epithelial cell; and 4) thickness of the alveolar wall (interstitial pneumonia). The severity of each criterion was scored from 0 to 3, which a high score indicating a severe histopathologic lesion. The sums of each criterion were used for the final score. Trachea and lung histopathologic changes were scored in their representative microscopic lesions.

### 2.14 Statistical analysis

Statistical analyses were performed using GraphPad Prism (Version 8.0.1, GraphPad Software). In the hamster experiments, the body weight change (%) or the sum of the histopathologic scores for each criterion was analyzed by a 2-tailed Mann-Whitney test.

## 3 Results

### 3.1 Construction of recombinant BoHV-4 expressing SARS-CoV-2 S glycoprotein

**Figure 1:**
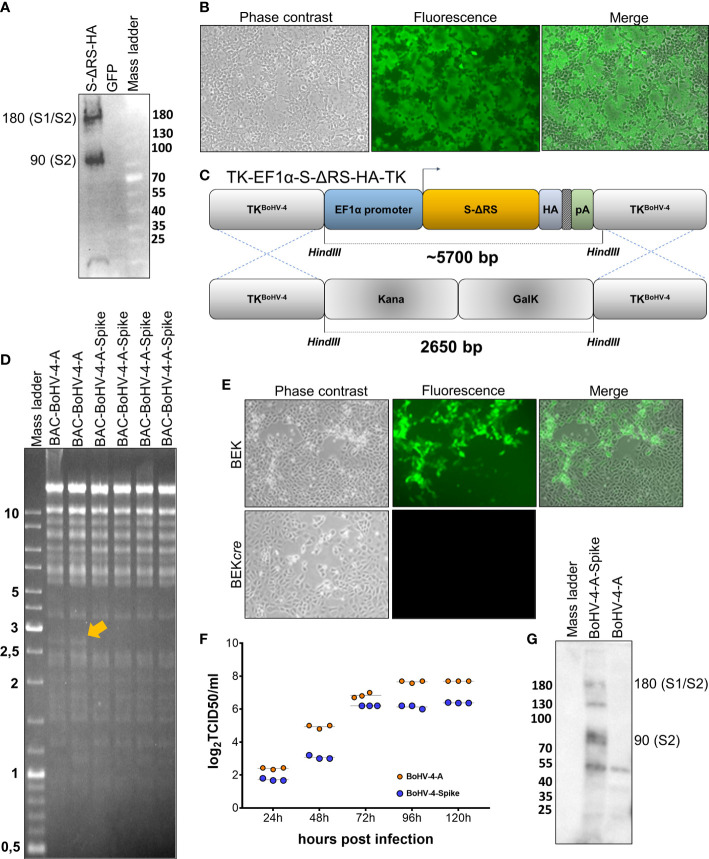
Generation and characterization of BoHV-4-A-S-ΔRS-HA-ΔTK (BoHV-4-A-Spike). **(A)** Western immunoblotting of pINT2-(TK-EF1α-S-ΔRS-HA-TK)-transfected HEK293T cell protein extract (S-ΔRS-HA; 40 µg) and pEGFP-C1-transfected HEK293T cell protein extract (GFP; 40 µg) employed as the negative control. **(B)** Representative microscopy images (phase contrast, fluorescence, and merged fields; 10X) of syncytia generated by the co-transfection of HEK/ACE2/TMPRRS2/Puro cells with the pINT2-(TK-EF1α-S-ΔRS-HA-TK) construct and pEGFP-C1. Diagram (not to scale; **(C)** showing the retargeting event obtained by heat-inducible homologous recombination in SW102 containing pBAC-BoHV-4-A-TK-KanaGalK-TK where the Kana/GalK cassette was replaced with EF1α-S-ΔRS-HA expression cassettes flanked by BoHV-4 TK sequences, located in a pINT2 shuttle plasmid vector [pINT2-(TK-EF1α-S-ΔRS-HA-TK)]. **(D)** Representative 2-deoxy-galactose-resistant colonies (pBAC-BoHV-4-A-Spike) tested by HindIII restriction enzyme analysis and agar gel electrophoresis and compared to the pBAC-BoHV-4-A parent. The 2,650 bp band (yellow arrow), corresponding to the un-retargeted pBAC-BoHV-4-A-TK-KanaGalK-TK control, was replaced with a ~5700 bp band in BoHV-4-A-S-ΔRS-HA-ΔTK (BoHV-4-A-spike) and not distinguishable due to overlapping with other bands of the same size. **(E)** Representative phase contrast images, fluorescent, and merged fields of plaques formed by viable reconstituted recombinant BoHV-4-A-S-ΔRS-HA-ΔTK (BoHV-4-A-Spike) after the corresponding BAC DNA was electroporated into BEK cells or BEK cells expressing *cre* recombinase (magnification, ×10). **(F)** Replication kinetics of BoHV-4-A-S-ΔRS-HA-ΔTK (BoHV-4-A-Spike) growth in BEK cells compared to the parental BoHV-4-A isolate. The data presented are the means ± standard errors of triplicate measurements (P > 0.05 for all time points as measured by the Student’s *t*-test). **(G)** Western immunoblotting of cells infected with BoHV-4-A-S-ΔRS-HA-ΔTK (BoHV-4-A-Spike). The lanes were loaded with 20 μg of protein extract. The negative control was established with BoHV-4-A-infected cells.

Recombinant BoHV-4 delivering SARS-CoV-2 S was constructed. Initially, a suitable expression cassette capable of driving the efficient expression of S was generated. The CoV-2 S open reading frame (ORF) sequence (https://www.ncbi.nlm.nih.gov/protein/1791269090) was depleted of its last 57 bp, coding for the endoplasmic reticulum retrieval signal (ERRS; peptide: *KFDEDDSEPVLKGVKLHYT*) (23) and substituted with a hemagglutinin (HA) tag. The designed ORF (S-ΔRS-HA) was human codon usage adapted with the Jcat codon adaptation tool (http://www.jcat.de), and chemically synthesized. S-ΔRS-HA was subcloned into a BoHV-4 pINT2 shuttle vector containing two BoHV-4 TK gene homologous sequences, an elongation factor 1α (EF1α) promoter, and a bovine growth hormone polyadenylation signal to generate pINT2-(TK-EF1α-S-ΔRS-HA-TK). pINT2-(TK-EF1α-S-ΔRS-HA-TK)-transfected HEK 293T cells successfully expressed S-ΔRS-HA **(** **Figure 1A** **)**. Further, when pINT2-EF1α-S-ΔRS-HA was cotransfected with pEGFP-C1, a construct expressing enhanced green fluorescent protein in a cell line stably expressing human ACE2 and TMPRSS2 (HEK/ACE2/TMPRRS2/Puro cells), large syncytia were observed **(** **Figure 1B**; **Supplementary Figure 1A** **)**, recapitulating the syncytiogenic activity of SARS-CoV-2 S during infection and was ascribable to the interaction with ACE2 and TMPRSS2. Next, BoHV-4-A-S-ΔRS-HA-ΔTK delivering the optimized EF1α-S-ΔRS-HA expression cassette was generated by heat inducible homologous recombination in an SW102 *E. Coli* strain containing pBAC-BoHV-4-A-KanaGalK-ΔTK **(** **Figure 1C** **)**. pBAC-BoHV-4-A-S-ΔRS-HA-ΔTK viral genome authenticity was first assessed by HindIII restriction enzyme analysis **(** **Figure 1D** **)** and then confirmed by Southern blotting using an S-ΔRS-HA-specific probe **(** **Supplementary Figure 1B** **)**. Clonal stability was ascertained by growing the positive clone over 20 passages (data not shown). BoHV-4-A-S-ΔRS-HA-ΔTK infectious viral particles were then obtained by BEK or BEK*cre* cell electroporation. BEK*cre* cells showed the depletion of the BAC/GFP cassette from the recombinant viral genome by the loss of green plaques **(** **Figure 1E** **)**. Moreover, BoHV-4-A-S-ΔRS-HA-ΔTK did not show strong replication defects compared to the BoHV-4-A parental strain **(** **Figure 1F** **),** and expressed S-ΔRS-H protein **(** **Figure 1G** **)**.

### 3.2 Assessment of BoHV-4-A-S-ΔRS-HA-ΔTK immunogenicity in mice

**Figure 2:**
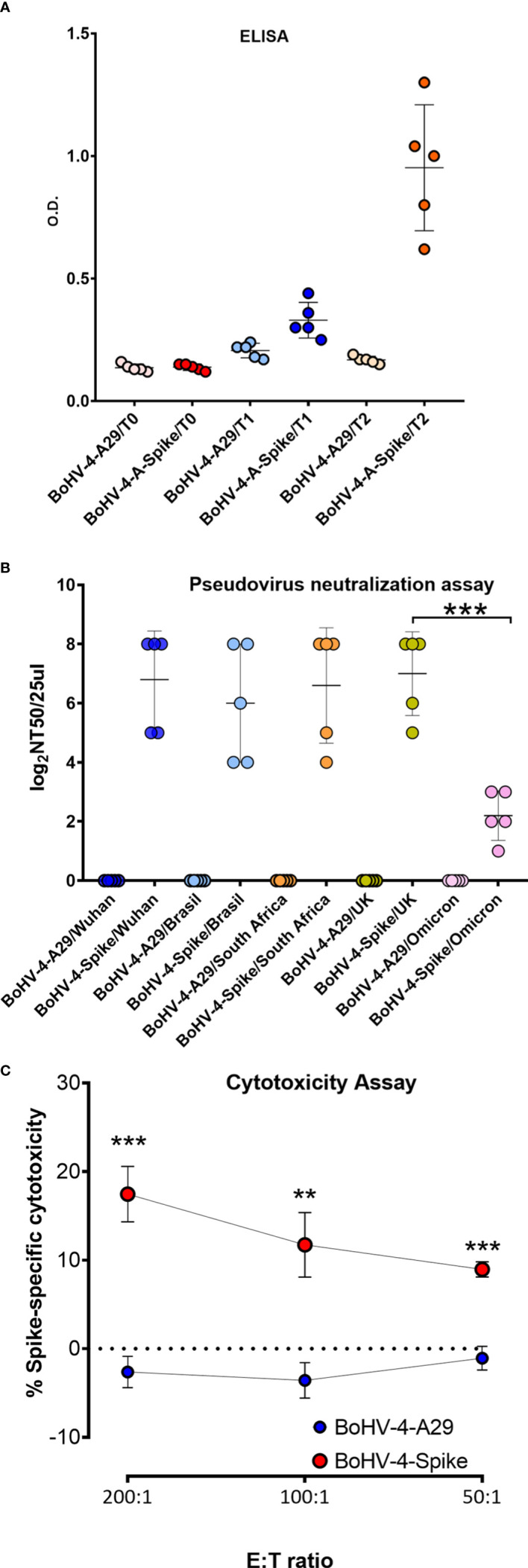
Evaluation of S antigen-specific antibody responses following immunization of mice with BoHV-4-A-S-ΔRS-HA-ΔTK (BoHV-4-A-Spike). **(A)** Mice were immunized with BoHV-4-A-S-ΔRS-HA-ΔTK (BoHV-4-A-Spike) and BoHV-4-A-CMV-A29ΔTK (BoHV-4-A-A29). ELISA tests assessing antigen-specific antibody responses. Mean data ± SEM are shown for each immunized group. **(B)** The same sera were tested for neutralizing serum antibodies by a pseudovirus neutralization assay against the 5 main variants. The 50% neutralization titer (NT_50_) value was the maximal dilution of the sera with signal reductions of ≥ 50%. **(C)** FACS of the percentage of 7-AAD^+^ dead cells among CFSE^+^ S-transfected 3T3/kB cells co-cultured for 48 hours with splenocytes from mice immunized with either BoHV-4-A29 or BoHV-4-Spike (N = 5 per group) at 200, 100 or 50:1 effector to target (E:T) ratios. The graph shows means ± SEM of the percentage of specific cell lysis. **P < 0.01; ***P < 0.001, Student’s *t*-test.

Before attempting a laborious and expensive challenge study with BoHV-4-A-S-ΔRS-HA-ΔTK in a high containment laboratory (BSL3), BoHV-4-A-S-ΔRS-HA-ΔTK immunogenicity was assessed in BALB/c mice. Two groups of mice (n = 5) were immunized (T0) with BoHV-4-A-S-ΔRS-HA-ΔTK or BoHV-4-A-A29, recombinant BoHV-4-A delivering an unrelated monkeypox virus antigen (16), and boosted 2 weeks apart (T1). Sera were collected from both groups of mice at T0 and T1. The mice were euthanized 2 weeks from T1 (T2), and serum and spleens were collected. Sera were tested by ELISA, and only the group of mice immunized with BoHV-4-A-S-ΔRS-HA-ΔTK showed the presence of antibodies against S protein **(** **Figure 2A** **)**. When the same sera were tested with a Pseudovirus Neutralization Assay, all BoHV-4-A-S-ΔRS-HA-ΔTK-inoculated mice produced serum neutralizing (SN) antibodies against the five variants tested, in contrast to BoHV-4-A-A29-inoculated mice **(** **Figure 2B** **).** However, SN antibody titers for the Omicron variant were significantly lower. Further, the ability of splenocytes to induce cytotoxicity in 3T3/KB cells expressing S protein was tested in an *in vitro* killing assay by co-culturing effector splenocytes from BoHV-4-A-S-ΔRS-HA-ΔTK and BoHV-4-A29-vaccinated mice. BoHV-4-A-S-ΔRS-HA-ΔTK-treated mice splenocytes displayed the enhanced lytic activity of S-expressing cells compared to BoHV-4-A29-treated mice splenocytes **(** **Figure 2C** **)**. No cytotoxic effect was observed in non-transfected 3T3/KB control cells (not shown). Therefore, this last data highlighted the activation of a specific cellular immune response pathway.

### 3.3 Monitoring of BoHV-4 lung transduction in mice by *in vivo* image analysis

**Figure 3:**
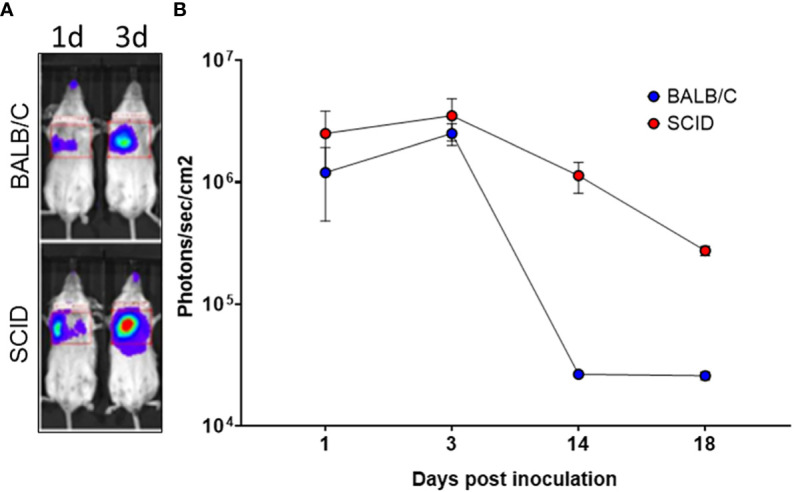
*In vivo* image analysis of mice intranasally inoculated with BoHV-4-A-CMVlucΔTK. **(A)** Representative *in vivo* bioluminescence images of SCID and BALB/c mice intranasally inoculated with 3×10^6^ TCID_50_ of BoHV-4-A-CMVlucΔTK. Luminescence signal was acquired 1, 3, 14, and 18 days post-intranasal inoculation of BoHV-4-A-CMVlucΔTK **(B)**.

Since one of the objectives of the investigation was to assess the IN route of BoHV-4-BV-mediated antigen delivery, it was of primary interest to know which part of the respiratory tract could be transduced by BoHV-4-BV and analyze the persistency of the antigen. For this end, a group (n = 5) of BALB/c and a group of SCID (n = 5) mice were intranasally inoculated with recombinant BoHV-4 delivering the luciferase expression cassette BoHV-4-A-CMVlucΔTK (26), and monitored by *in vivo* image analysis 1, 3, 14, and 18 days post-inoculation. In both groups of mice, the majority of transduction was observed in the lungs as fast as 1-day post-inoculation, peaked on day 3, and declined progressively **(** **Figure 3A, B** **)**. As expected, the signal decline was faster in BALB/c mice than in SCID mice, and this was probably due to the involvement of the immune system.

**Figure 4:**
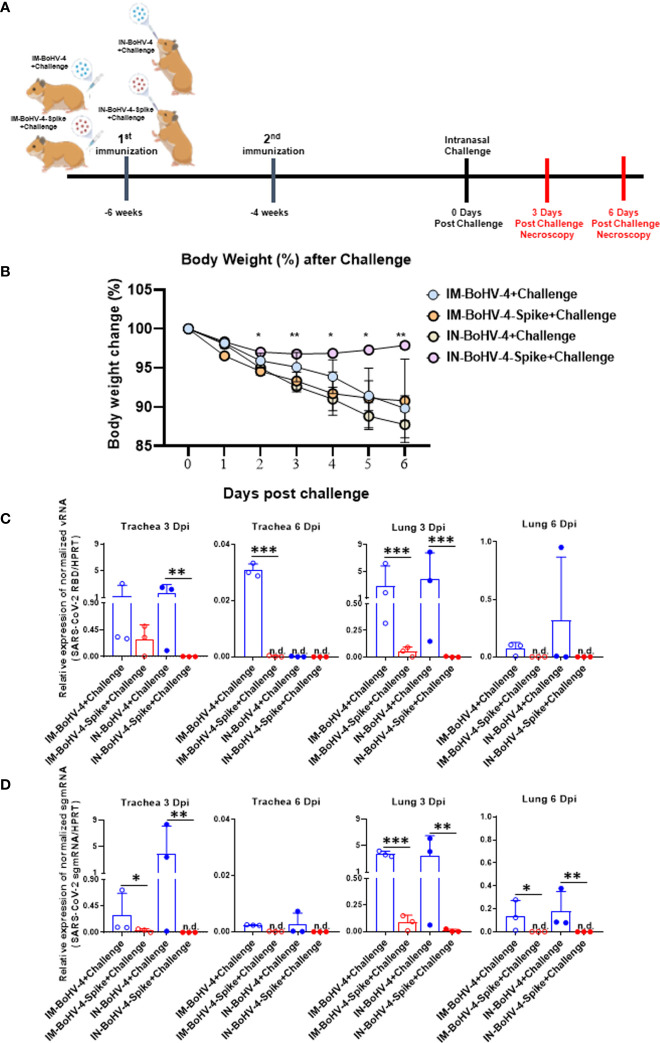
Intranasal administration of BoHV-4-A-S-ΔRS-HA-ΔTK reduces body weight loss and viral load in hamster tracheas and lungs following SARS-CoV-2 challenge. Five-week-old male Syrian golden hamsters were intramuscularly or intranasally inoculated twice with BoHV-4 vector delivering the Spike protein of SARS-CoV-2 or control vector vaccine candidate. Six weeks after the first vaccination, hamsters were intranasally challenged with 4 x 10^5^ TCID_50_/hamster SARS-CoV-2. **(A)** Groups and experimental challenge scheme. **(B)** Body weight. Changes in average animal weight relative to weight at 0 dpi (mean ± SD). **(C, D)** SARS-CoV-2 viral qPCR results. Trachea and lungs were harvested from each group at 3 and 6 dpi (mean + SD). n.d., not detected. *P < 0.05, **P < 0.01, ***P < 0.001, Mann-Whitney test.

### 3.4 Intranasal administration of BoHV-4-A-S-ΔRS-HA-ΔTK reduces body weight loss and viral load in hamster trachea and lung following SARS-CoV-2 challenge

The hamster SARS-CoV-2 challenge model was exploited to assess the efficacy of IN vaccination in an animal model of infectious lung disease. In accordance with the animal experimentation ethics committee, which strongly suggests limiting animal life waste and suffering as much as possible, the number of animals per group was limited to 3 (n = 3). Two groups of animals were inoculated *via* the IM route (IM-BoHV4-Spike and IM-BoHV4-control group), and 2 groups of animals were inoculated *via* the IN route (IN-BoHV4-Spike and IN-BoHV4-control). All 4 groups of animals received a prime-boost regimen and were challenged 4 weeks after the boost with a pathogenic dose of SARS-CoV-2. IM-BoHV-4-A-Spike, IM-BoHV-4-A, IN-BoHV-4-A-Spike, and IN-BoHV-4-A hamsters were euthanized 3 or 6 days post-challenge **(** **Figure 4A** **)**. After the challenge and before euthanasia, all animals were monitored daily for body weight change. No significant body weight change was observed for animals in the IN-BoHV-4-A-Spike groups, whereas the other groups of animals progressively lost body weight **(** **Figure 4B** **)**. RT-PCR for SARS-CoV-2 viral gRNA (RBD) and sgmRNA (N) 3 and 6 dpi revealed reductions mainly in the lungs of hamsters in the IM-BoHV-4-A-Spike and IN-BoHV-4-A-Spike-treated groups **(** **Figures 4C, D** **)**. Hence, vaccination with BoHV-4-A-Spike induced SARS-CoV-2 reductions in the lungs independently of the administration route and when administered intranasally, significantly prevented clinical manifestations of the disease, represented by weight loss.

### 3.5 Intranasal administration of BoHV-4-A-S-ΔRS-HA-ΔTK significantly reduced lung lesions induced by SARS-CoV-2 challenge in hamsters

**Figure 5:**
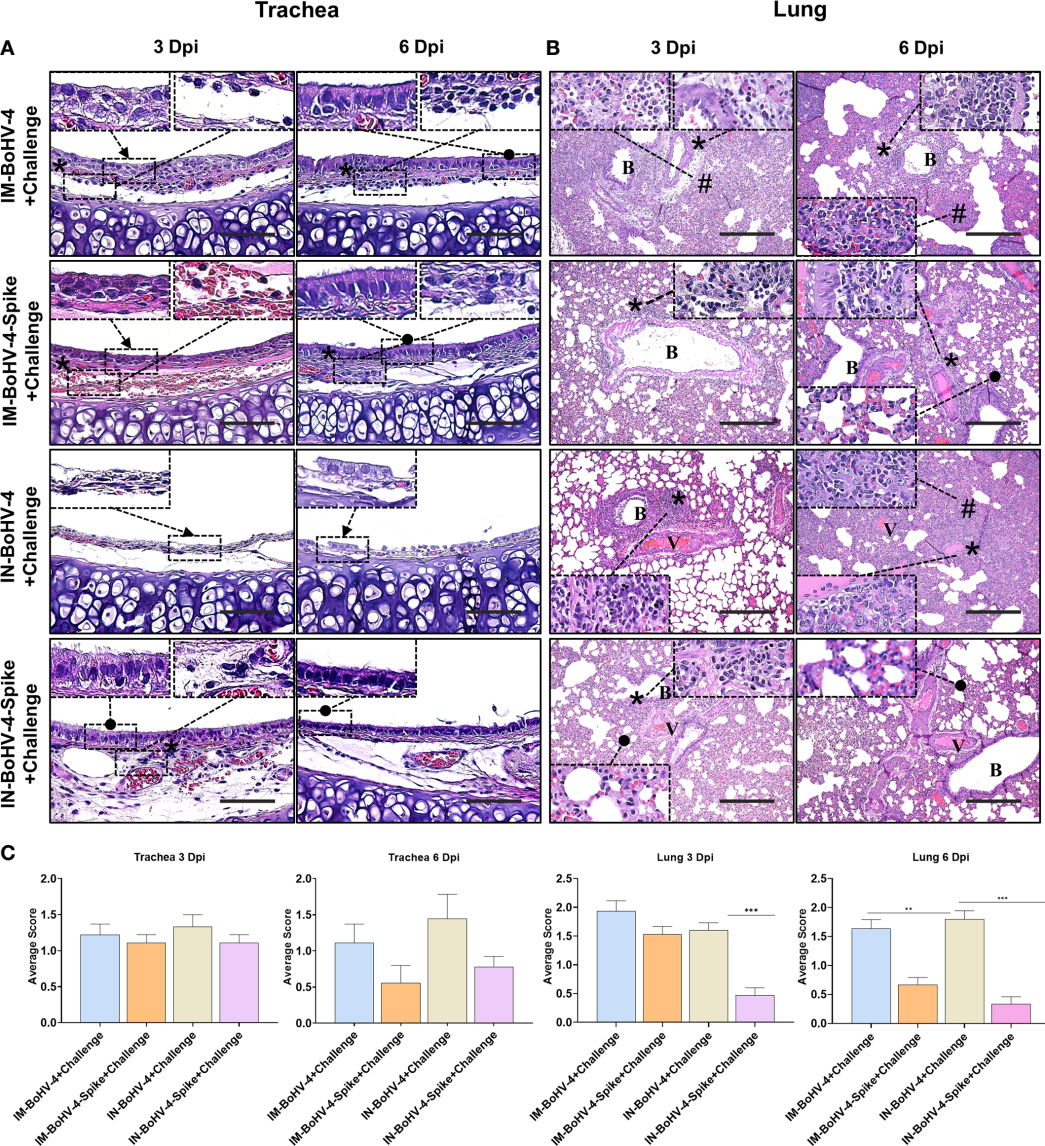
Intranasal administration of BoHV-4-A-S-ΔRS-HA-ΔTK significantly reduced lung lesions induced by SARS-CoV-2 challenge in Hamsters. **(A)** Representative pictures of trachea sections. Non-ciliated cuboidal and/or squamous cells (black arrow), which were transformed from normal ciliated columnar epithelial cells, were mainly observed in both the IM and IN-BoHV-4-A-vaccinated groups at 3 dpi. Ciliated columnar epithelial cells (black dots) were detected in the IN-BoHV-4-Spike group at 3 dpi and the IM groups at 6 dpi. Mononuclear inflammatory cells infiltrated (asterisks) into the lamina propria. H&E, scale bar, 50 μm; Insert: higher magnification of the indicated regions. **(B)** Representative lung section images. Moderate to severe mononuclear inflammatory cell infiltration into the peribronchiolar (asterisks) and/or alveolar wall (harsh) in the IM groups and IN-BoHV-4 group at 3 dpi. However, the IN-BoHV-4-Spike group showed not only less mononuclear inflammatory cell infiltration in peribronchiolar regions but also normal pneumocytes (black dot) at 3 dpi. At 6 dpi, the IM and IN control groups showed moderate interstitial pneumonia (asterisks, inflammation in peribronchiolar regions; harsh, thickening of the alveolar wall with inflammation). Few inflammatory cell infiltrations were observed in the peribronchiolar regions (asterisks) in the IM-BoHV-4-A group, however, most pneumocytes were normally observed (black dots). No obvious pathologic changes were observed in the IN-BoHV-4-A-Spike group compared to the IN-control group. H&E; B, bronchiole; V, vessel. Scale bars, 200 μm; Insert: higher magnification of the indicated regions. **(C)** Histopathology scores of the tracheas and lungs. Data are presented as means + SD. ***P < 0.001, Mann-Whitney test. The mean histopathological scores in tracheal lesions of the IM-spike-challenge and IN-Spike-challenge groups at 6 dpi were lower than that of the IM-control-challenge and IN-control-challenge groups, respectively, but without statistical difference. However, the mean histopathological scores of lung lesions in the IN-Spike-challenge group were significantly (P < 0.0001) lower than those in the IN-BoHV-4+challenge group at 3 and 6 dpi.

Histopathologic scores of lesions in the tracheas and lungs of the 8 groups of hamsters were compared. At 3 dpi, non-ciliated cuboidal and/or squamous cells, which were turned from normal ciliated columnar epithelial cells, were mainly observed in both the IM and IN-BoHV-4-A-vaccinated groups **(** **Figure 5A** **)**. In the IN-BoHV-4-A-Spike group, a relatively high frequency of epithelial cells was detected as ciliated columnar epithelial cells. Notably, the IN-BoHV-4-A-Spike group showed relatively improved tracheal epithelial lesions at all tested dpis, and mononuclear inflammatory cell infiltration in the lamina propria was observed at 3 dpi. However, no significant scoring differences in the trachea were seen based on our histopathological criteria (above mentioned trachea histopathologic criteria 2. Inflammatory cell infiltration of the lamina propria, **Figure 5C**). The IN-BoHV-4-A-Spike group showed a reduced mononuclear inflammatory cell infiltration in the peribronchiolar lung regions with respect to the IN-BoHV-4-A control group and both IM groups, which showed a moderate to severe inflammatory cell infiltration in the same regions at 3 dpi **(** **Figure 5B** **)**. Additionally, the IM-BoHV-4-A group showed mononuclear inflammatory cell infiltration into the alveolar wall. Therefore, only the IN-BoHV-4-A-Spike group showed significantly lower histopathological scores compared to their control group at 3 dpi **(** **Figure 5C** **)**. At 6 dpi, moderate interstitial pneumonia, which included alveolar wall thickening, inflammatory cell infiltration into the alveolar wall, and peribronchiolar mononuclear inflammatory cell infiltration was observed in the IM and IN control groups. Few mononuclear inflammatory cells infiltrated the peribronchiolar regions in the IM-BoHV-4-A-Spike group. However, most of the pneumocytes observed did not have alterations. No obvious pathologic changes were observed in the IN-BoHV-4-A-Spike group compared to the IN-control group. Moreover, no turbinates’ histological lesions were observed in all IN-inoculated animals **(** **Supplementary Figure 2** **)**. Collectively, these data demonstrated that intranasally BoHV-4-A-Spike-vaccinated hamsters showed considerable protection from SARS-CoV-2 infections, as evidenced by a reduction in body weight loss and improvements in lung histopathological lesions during the early infection period.

## 4 Discussion

Respiratory infections in humans and animals are one of the main causes of death worldwide. Thus, the appearance of new respiratory infectious diseases remains a concern for human and animal health (27). Vaccines are a valuable tool for disease prevention and, in many cases, have been able to reduce disease incidence, thus limiting mortality and morbidity (28). Traditionally, vaccines were mainly represented by dead or attenuated microorganisms or by their purified antigens and administered by IM injections (28). The COVID-19 pandemic introduced the use of viral vector or mRNA-based vaccines to clinics and prompted the experimentation of other administration routes. Indeed, vaccines administered by IM injection can induce a systemic immune response that protects from disease symptoms and severity, but in many cases, are not able to prevent infection and pathogen transmission in the population since they fail to induce substantial mucosal immunity (29–31). The nasal cavity is the first contact with inhaled pathogens that cause mucosal-transmitted disorders. Therefore, vaccine administration through the nasal route is a promising and cost-effective approach to prevent the spread of a respiratory-transmitted pathogen. Indeed, IN vaccination could combine better patient compliance with higher efficiency in activating mucosal immunity while maintaining the ability to induce systemic humoral and cellular immune responses (32). IN vaccination using a SARS-CoV-2 Spike protein-based chimpanzee adenovirus-vectored vaccine was shown to induce a long-lasting mucosal immune response able to limit airway infection in different animal models of SARS-CoV-2 infection, including BALB/c mice, mice transgenic for the human ACE2 Spike receptor, hamsters, and non-human primates (33–36). This viral vector-based vaccine, as well as other intranasally-administered adenovirus-based vaccines and vaccines based on recombinant Spike protein or viral subunits, is currently undergoing clinical trials (37). Since BoHV-4-BV has been shown to have good characteristics as a platform for animal vaccination and potentially for non-human primates and human beings, it was of interest to test its protective efficacy following IN administration in a well-established model of respiratory tract virally induced disease, the COVID-19/Syrian hamster model (38). Recombinant BoHV-4, BoHV-4-A-S-ΔRS-HA-ΔTK, delivering the unstable form of SARS-CoV-2 Spike glycoprotein, was constructed and characterized. Although the mutated stabilized Spike form was shown to elicit stronger immunity with respect to the unstable Spike form, the latter was chosen to stress the characteristics of the delivery system rather than those of the antigen employed. However, the syncytiogenic activity of Spike delivered by BoHV-4-A-S-ΔRS-HA-ΔTK was well maintained, as well as its capacity to interact with ACE2/TMPRSS2 and generate pseudovirus neutralizing antibodies in the serum (data not shown). Further, the cytoplasmic tails of SARS-CoV-2 Spike glycoprotein contain an endoplasmic reticulum retrieval signal (ERRS) that can retrieve Spike proteins from the Golgi to the endoplasmic reticulum (ER). This process is thought to accumulate Spike proteins at the SARS-CoV-2 budding site, the ER-Golgi intermediate compartment (ERGIC). Therefore, the Spike-protein cytoplasmic tail was truncated to facilitate Spike-protein incorporation into the Golgi apparatus and increase cell surface expression levels (23, 39).

The safety and immunogenicity of BoHV-4-A-S-ΔRS-HA-ΔTK were initially tested in BALB/c mice through intraperitoneal inoculation to show delivery system functionality in an already established experimental setting. Mice showed that a prime and boost vaccination strategy strongly stimulated the production of neutralizing antibodies against different viral variants of concern. A lower titer of antibodies capable of binding to the Omicron variant compared to others was expected, as it was observed for most vaccines targeting the Wuhan Spike glycoprotein (40). Of note, vaccination elicited T-cell cytotoxicity of Spike-expressing cells and several data have highlighted the importance of T cells in mediating immunity to SARS-CoV-2 and protecting from severe disease (41). Moreover, although BoHV-4-BV was intranasally administered in cattle in a previous study, no challenge with a specific pathogen was performed to test its immunogenicity (42) and because our final purpose was to evaluate the potential protective efficacy of a candidate antigen delivered by BoHV-4-BV *via* the respiratory tract, it was of interest to know if BoHV-4-BV could transduce the lungs of mice. To this end, a whole animal bioluminescent imaging approach that included BoHV-4 delivering the enzyme luciferase (26) and BoHV-4-A-CMVlucΔTK, was adopted. Whole animal bioluminescent imaging (BLI) is extensively applied in the analyses of many *in vivo* cellular events because of its low cost, high throughput, and simplicity of performing. Further, it allows monitoring a single individual, decreasing the inter-animal variation, increasing resolution, and reducing data loss (43, 44). Having an integrated bioluminescent reporter gene in the viral genome not only allows the rapid quantification of viral replication levels but also, upon introduction of the luciferase substrate, provides noninvasive imaging of infected tissues. In fact, dynamic whole-body imaging of living animals allows assessments not only of where the infection starts in the body but also where it spreads.

The main concern about BoHV-4 IN administration was its safety in terms of neurotropism and potential neuropathogenicity, as BoHV-4 was previously shown to be able to transduce cells of the rostral migratory stream and ependyma in the absence of specific neurological symptoms when stereo-tactically inoculated into the lateral ventricles of adult mice brains (6). The nasal mucosa represents a direct route to get inside the central nervous system (CNS). Compounds, as well as neurotropic viruses, can reach the olfactory bulb *via* retrograde transport or be absorbed into the submucosa and gain the cerebrospinal fluid (45). The attenuated parainfluenza virus vaccine can infect olfactory neurons and gain access to the CNS (46, 47). One of the influenza virus infection complications in children is encephalitis induced by viral replication in the olfactory neuroepithelium and its transport to the CNS, resulting in glial activation and neuroinflammation (48). SARS-CoV-2 has been detected in the brain of some deceased COVID-19 patients, and mouse studies demonstrated the migration of the virus from the olfactory nerve to distal neurons (49, 50). Further, herpes simplex virus 1 (51), as well as poliovirus (52), have a common route of entry into the olfactory bulb. Therefore, live attenuated viral vaccines and viral vectors must be evaluated carefully in terms of neurovirulence if intended to be administered intranasally. However, IN-inoculated animals did not show a specific behavior attributable to a potential neurovirulence of the vector, during the observation period. This is an issue that requires further investigation.

Although BoHV-4-BV was replication-competent *in vitro* and in hamster cells, it was not the case *in vivo*, even when inoculated intranasally or intramuscularly. However, IN administration was able to generate higher protection than IM administration. This was probably because the IN delivery of an antigen produces potent protective systemic immunity, due at least in part to the fact that the upper respiratory mucosa is rich in lymphoid tissue and functional antigen-presenting dendritic cells (53). Moreover, IN immunization has other advantages. First, the noninvasive nature and ease of administration of IN vaccines facilitate widespread vaccine implementation (53). Lastly, IN vaccines are needle-free and, thus, lowering iatrogenic pathogen transmission risk. These characteristics of IN vaccines strongly increase their field-use potential in livestock production management. In conclusion, the data described in the present work support the use of BoHV-4-BV as a safe, large-capacity viral vector, administrable intranasally in animals.

In the second part of the experiment, we demonstrated that IN BoHV-4-A-S-ΔRS-HA-ΔTK vaccines provided greater protection against SARS-CoV-2 infection in a Syrian golden hamster model, compared to IM vaccines. The IN-vaccinated group showed improved clinical signs, including significantly less body weight loss and less viral load in the trachea and lungs. Although the histopathologic trachea scores were not significantly different, which was due to the inflammatory cell infiltration criteria, histopathologic lung scores were significantly lower in the IN-vaccinated group on all tested dpis. The findings suggest that the mechanism underlying IN immunization with our vaccine may be attributed to the development of mucosal immunity, which might have a more important role in SARS-CoV-2 infection than systemic immunity (54). SARS-CoV-2 is known as a typical mucosal pathogen that can infect human epithelial cells in the respiratory tract (54). In natural infection, SARS-CoV-2 infects respiratory epithelial cells of the upper respiratory regions, and then the virus reaches lower lung airways (54). Therefore, mucosal immunity, consisting of innate immunity, including phagocytic neutrophils, dendritic cells (DCs), natural killer cells, and macrophages, and adaptive immunity, characterized by mucosal secretory IgA (sIgA) antibodies and resident memory T (TRM) cells, is an initial defense against SARS-CoV-2 infection (55). Recent studies demonstrated that IN vaccines effectively induced sIgA secretions (56) and TRM generation (33) compared to IM vaccines. Similar results were observed in another IN SARS-CoV-2 viral vector vaccine study. Replication-defective adenovirus (Ad) 5-vectored vaccine encoding full-length Spike protein increased IgA antibodies in trachea-lung washes by only IN administration (57). IN immunization with chimpanzee Ad-vectored SARS-CoV-2 vaccine (ChAd-SARS-CoV-2-S) increased the percentage of IFNγ-secreting CD8^+^ TRM cells in the lungs and induced long-term immunity and excellent protection against mutated strains (33) (B.1.351, B.1.1.28 and B.1.617.1). Moreover, IN immunization using ChAdOx1 (an Ad-based vaccine encoding the Spike protein of SARS-CoV-2) reduced the nasal shedding of SARS-CoV-2 and showed complete protection in a co-housed transmission experiment using hamsters compared to IM vaccination, which only reduced viral lung titers (58). Currently, all approved COVID-19 vaccines are administered through IM injections. Pre-existing immunity to the viral vector is a common concern for IM vaccinations, and high pre-existing immunity weakened humoral and cellular immune responses in some clinical trials (59). However, IN vaccination has the potential to confer immunity without influence by pre-existing immunity (60). Therefore, administering additional booster doses through IN vaccination may be an effective way to boost mucosal immunity (61).

It should be noted that our hamster experiments had some limitations. First, the detailed mechanism of action of our BoHV-4 vaccines was not elucidated. We explored two different time points to have a clearer picture of the evolution of the pathological signs and immune infiltration, but due to animal number limitations, this led to a small sample size that was not conducive to rigorous statistical analysis. Second, IN challenge with the SARS-CoV-2 virus is considered an efficient challenge route, but a high dose of live viruses is directly sent to the upper and lower respiratory regions. In contrast, in natural SARS-CoV-2 infection situations, viral infection is low-dose but persistent (62). Therefore, the protective efficacy of our vaccine should be demonstrated in other experiment designs, such as a transmission model. Third, it should be noted that the limited number of animals in our study hindered the attainment of robust statistical significance in the quantification of vRNA and sgmRNA. Fourth, it is plausible that the nose or nasal turbinates, which we found to be not affected, at least histologically, may provide a more accurate depiction of the infection dynamics in SARS-CoV-2. Finally, as new COVID-19 virus variants of concern (VOCs), which could evade the defenses of existing vaccines, continue to emerge, there is a need for additional studies on protective effects against different VOCs, as well as studies on long-term immunity. In conclusion, the data described in the present work support the use of BoHV-4-BV as a safe, large-capacity viral vector, administrable intranasally in animals.

## Data availability statement

The original contributions presented in the study are included in the article/**Supplementary Material.** Further inquiries can be directed to the corresponding authors.

## Ethics statement

The animal study was reviewed and approved by The European guidelines, Directive 2010/63, and with the approval of the Animal Care and Use Committee of the University of Torino and the Italian Ministry of Health. The hamster studies, including challenging and necropsy, were performed in an animal biosafety level 3 (ABL3) facility of the Korea Zoonosis Research Institute (KOZRI) at Jeonbuk National University, with approval from the Institutional Animal Care and Use Committee (JBNU 2022-049).

## Author contributions

GD and BK conceptualized the study. GD and BK designed the experiments with the contributions of S-CP and LC. GD and BK wrote the paper with the contributions of LC and S-CP. S-CP, LC, VF, BO, M-SY, GH, AD, EB, FC, and GD performed the experiments and analyzed the data. GD and BK coordinated and directed the study. All authors contributed to the article and approved the submitted version.
