# Supplementary material for: Assessment of BoHV-4-based vector vaccine intranasally administered in a hamster challenge model of lung disease
Source: Front Immunol. 2023 Jul 6;14:1197649. doi: 10.3389/fimmu.2023.1197649 (PMC10358724; doi:10.3389/fimmu.2023.1197649)
Supplement: Supplementary file 1 [file DataSheet_1.pdf]

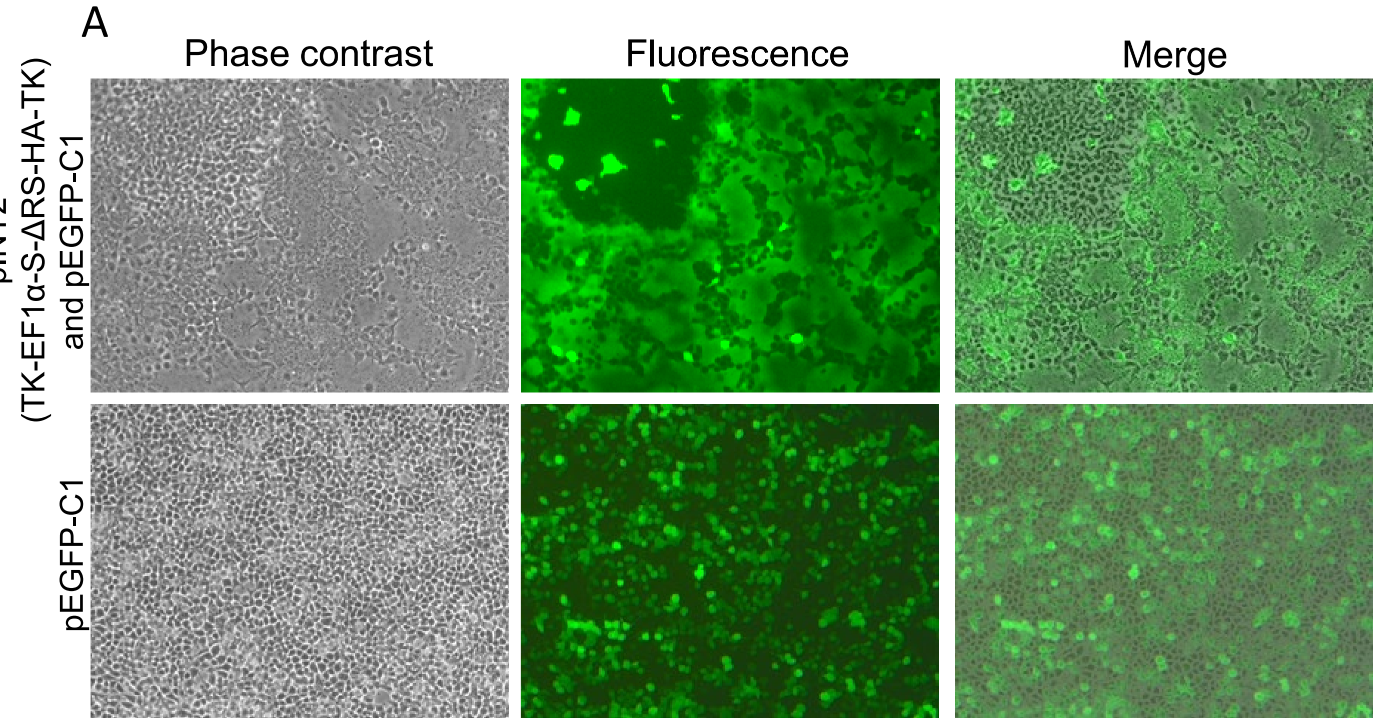

A). Representative microscopic image (Phase contrast, Fluorescence and Merged fields; 10X) of HEK/ACE2/TMPRRS2/Puro cells cotransfected with pINT2-(TK-EF1α-S-ΔRS-HA-TK) construct and pEGFP-C1 or only pEGFP-C1. Large syncytia were observed only in cells transfected with pINT2-(TK-EF1α-S-ΔRS-HA-TK) construct and pEGFP-C1.

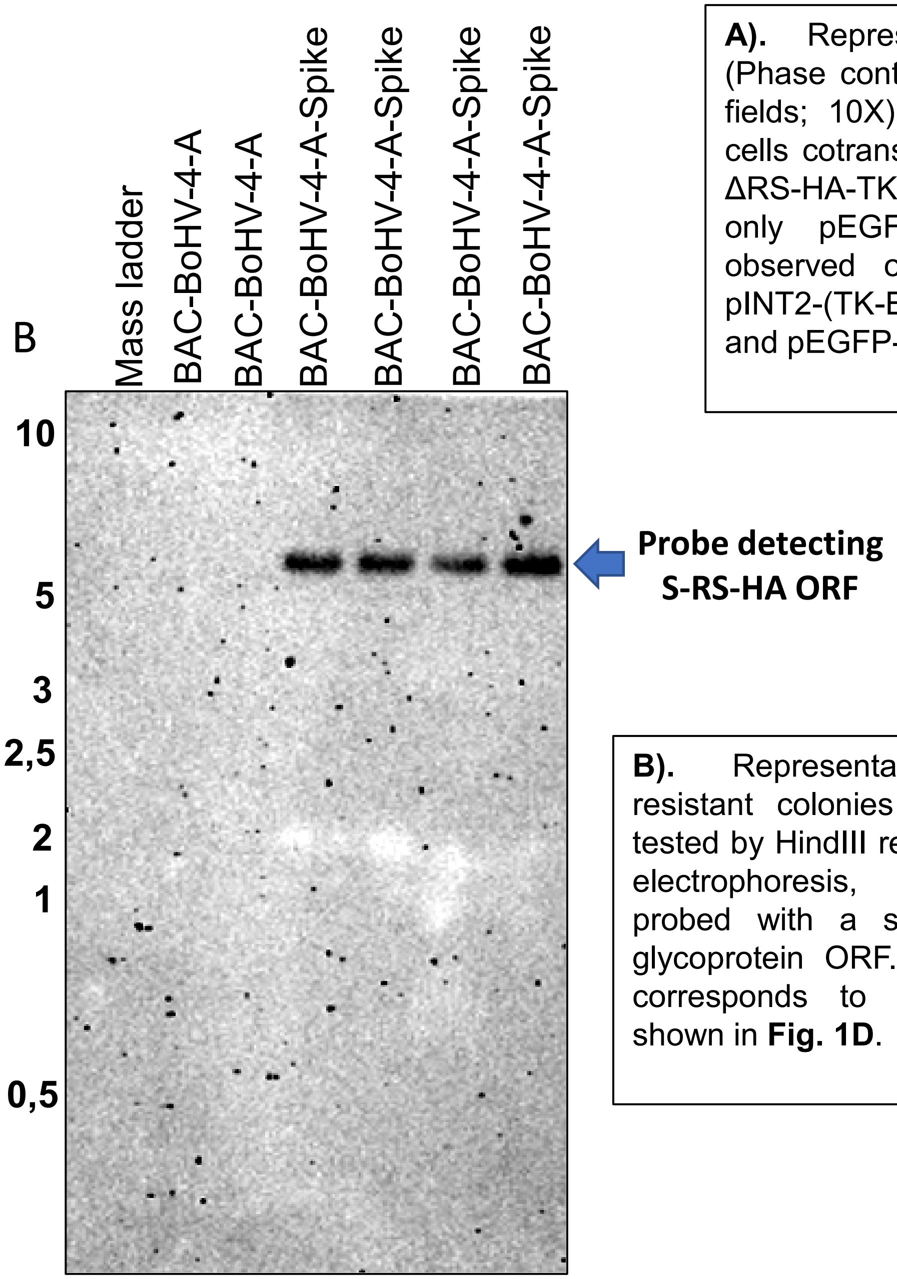

B). Representative 2-deoxy-galactose resistant colonies (pBAC-BoHV-4-A-Spike) tested by HindIII restriction enzyme analysis electrophoresis, southern blotting and probed with a specific probe for Spike glycoprotein ORF. This southern blotting corresponds to the gel electrophoresis shown in **Fig. 1D**.
