# Supplementary material for: Assessment of BoHV-4-based vector vaccine intranasally administered in a hamster challenge model of lung disease
Source: Front Immunol. 2023 Jul 6;14:1197649. doi: 10.3389/fimmu.2023.1197649 (PMC10358724; doi:10.3389/fimmu.2023.1197649)
Supplement: Supplementary file 2 [file DataSheet_2.pdf]

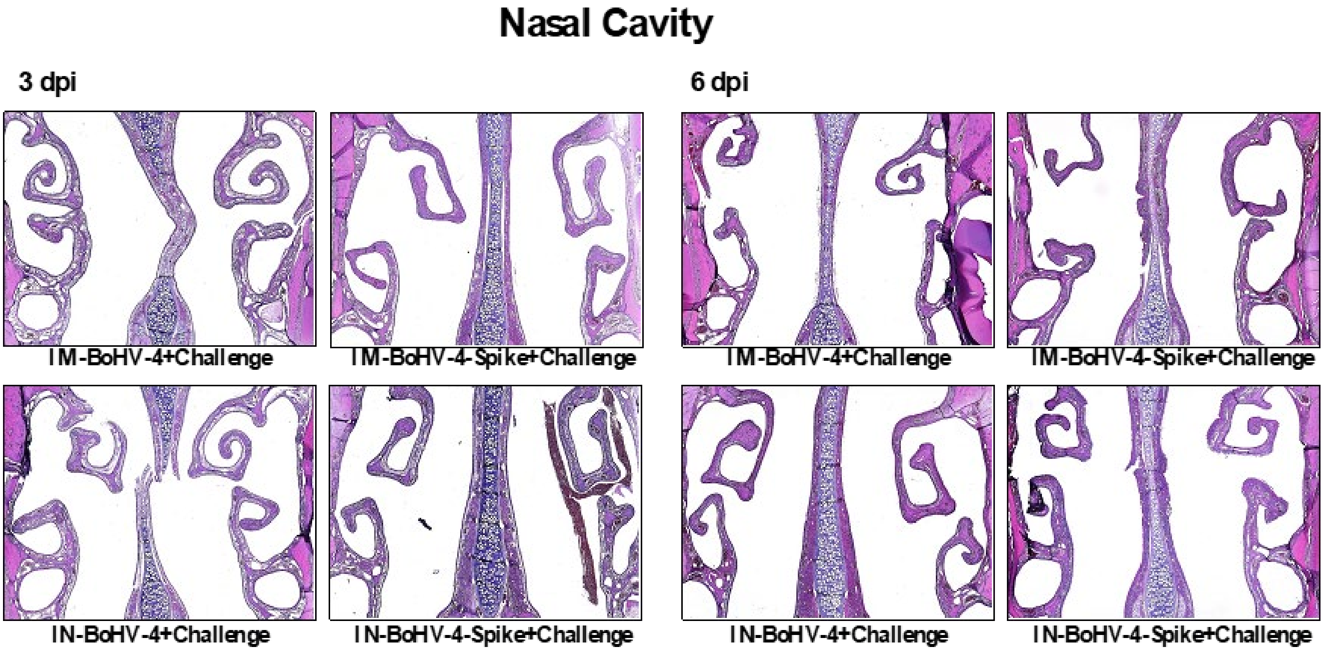

H & M Representative pictures of turbinates sections.
At different time post vaccination and challenge.
